# Striatal DAT and extrastriatal SERT binding in early-stage Parkinson's disease and dementia with Lewy bodies, compared with healthy controls: An ^123^I-FP-CIT SPECT study

**DOI:** 10.1016/j.nicl.2019.101755

**Published:** 2019-03-12

**Authors:** Merijn Joling, Chris Vriend, Pieter G.H.M. Raijmakers, Jessica J. van der Zande, Afina W. Lemstra, Henk W. Berendse, Jan Booij, Odile A. van den Heuvel

**Affiliations:** a Amsterdam UMC, Vrije Universiteit Amsterdam, Neurology, Amsterdam Neuroscience, De Boelelaan 1117, Amsterdam, Netherlands; b Amsterdam UMC, Univ of Amsterdam, Radiology and Nuclear Medicine, Amsterdam Neuroscience, Meibergdreef 9, Amsterdam, Netherlands; c Amsterdam UMC, Vrije Universiteit Amsterdam, Psychiatry, Amsterdam Neuroscience, De Boelelaan 1117, Amsterdam, Netherlands; d Amsterdam UMC, Vrije Universiteit Amsterdam, Anatomy and Neurosciences, Amsterdam Neuroscience, De Boelelaan 1117, Amsterdam, Netherlands; e Amsterdam UMC, Vrije Universiteit Amsterdam, Radiology and Nuclear Medicine, Amsterdam Neuroscience, De Boelelaan 1117, Amsterdam, Netherlands; f Amsterdam UMC, Vrije Universiteit Amsterdam, Neurology, Alzheimer Center, Amsterdam Neuroscience, De Boelelaan 1117, Amsterdam, Netherlands

**Keywords:** Parkinson's disease, Dementia with Lewy bodies, ^123^I-FP-CIT, SERT, DAT

## Abstract

Parkinson's disease (PD) and dementia with Lewy bodies (DLB) are thought to be part of a spectrum: both have a clinical profile including symptoms associated with dopaminergic and serotonergic loss, yet few imaging studies have focused on serotonergic neurodegeneration in both disorders. We aimed to study degeneration of terminals with dopamine and serotonin transporter (DAT and SERT, respectively) in patients with early-stage PD and DLB relative to healthy controls, using ^123^I-N-ω-fluoropropyl-2β-carbomethoxy-3β-(4-iodophenyl)nortropane (^123^I-FP-CIT) single photon emission computed tomography (SPECT).

We conducted region of interest (ROI) and voxel-based analyses on ^123^I-FP-CIT SPECT scans. Using the cerebellum as a reference region, we determined binding ratios (BRs) for bilateral ROIs in the DAT-rich striatum (head of the caudate nucleus and posterior putamen) and SERT-rich extrastriatal brain regions (thalamus, hypothalamus and hippocampus). We compared BRs in PD and DLB patients with BRs in healthy controls (all groups: *n* = 16).

Both PD and DLB patients had lower striatal ^123^I-FP-CIT BRs than healthy controls for the bilateral caudate head (PD—left: *F*(1,29) = 28.778, *P* < .001, *ω*^*2*^ = 0.35; right: *F*(1,29) = 35.338, *P* < .001, *ω*^*2*^ = 0.42; DLB—left: *F*(1,29) = 28.241, *P* < .001, *ω*^*2*^ = 0.31; right: *F*(1,29) = 18.811, *P* < .001, *ω*^*2*^ = 0.26) and bilateral posterior putamen (PD—left: *F*(1,29) = 107.531, *P* < .001, *ω*^*2*^ = 0.77; right: *F*(1,29) = 87.525, *P* < .001, *ω*^*2*^ = 0.72; DLB—left: *F*(1,29) = 39.910, *P* < .001, *ω*^*2*^ = 0.48; right: *F*(1,29) = 26.882, *P* < .001, *ω*^2^ = 0.38). DLB patients had lower hypothalamic ^123^I-FP-CIT BRs than healthy controls (*F*(1,29) = 6.059, *P* = .020, *ω*^*2*^ = 0.12). In the voxel-based analysis, PD and DLB patients had significantly lower striatal binding than healthy controls.

Both PD patients in the early disease stages and DLB patients have reduced availability of striatal DAT, and DLB patients lower hypothalamic SERT compared with healthy controls. These observations add to the growing body of evidence that PD and DLB are not merely dopaminergic diseases, thereby providing additional clinicopathological insights.

## Highlights

• ^123^I-FP-CIT binding is lower in the hypothalamus of DLB patients, compared with healthy controls, but there is no difference in ^123^I-FP-CIT binding in the thalamus and hippocampus between these groups.
• There is no difference in ^123^I-FP-CIT binding between early-stage PD patients and healthy controls in the SERT-rich thalamus, hypothalamus and hippocampus.
• This observation adds to the growing body of evidence that PD and DLB are not merely dopaminergic diseases.

## 1 Introduction

Parkinson's disease (PD) and dementia with Lewy bodies (DLB) are both neurodegenerative alpha-synucleinopathies. They are thought to be part of a clinical spectrum with overlapping symptoms, but to have a different disease course. The latter is the basis for the *one year rule* to clinically diagnose DLB, in which cognitive decline needs to predate the motor symptoms, or appear no less than one year thereafter (McKeith et al., 2005). A clinical diagnosis of PD by definition requires the presence of the classical motor symptoms, collectively called *parkinsonism* (Gibb and Lees, 1988*;* Hughes et al., 1992). Although parkinsonism is also frequently observed in DLB, symptoms such as hallucinations and dementia are more predominant (McKeith et al., 2005).

The clinical profile of both PD and DLB includes sleep disturbances, anxiety, depression, hallucinations, cognitive deterioration (for reviews, see (Politis and Niccolini, 2015; Ferrer et al., 2012)), and also autonomic symptoms such as orthostatic hypotension (Thaisetthawatkul et al., 2004; Andersson et al., 2008). The broadness of these clinical profiles implies that the pathophysiology of both alpha-synucleinopathies involves a dysfunction of a variety of neurotransmitter systems. Indeed, there is evidence for degeneration of multiple neurotransmitter systems in both diseases, including the well-known degeneration of the dopaminergic system (Piggott et al., 1999), but also of serotonergic (Roselli et al., 2010; Azmitia and Nixon, 2008) and cholinergic systems (Hepp et al., 2013).

In both PD and DLB, degeneration of the nigrostriatal dopamine system has been demonstrated using ^123^I-N-ω-fluoropropyl-2β-carbomethoxy-3β-(4-iodophenyl)nortropane (^123^I-FP-CIT) single photon emission computed tomography (SPECT) (Walker et al., 2004; O'Brien et al., 2004). This radiotracer has a high affinity for the presynaptic dopamine transporter (DAT) (Booij et al., 1997), and additionally a modest affinity for the presynaptic serotonin transporter (SERT) (Abi-Dargham et al., 1996). Therefore, it is possible to simultaneously use ^123^I-FP-CIT as a proxy for the integrity of both the striatal dopaminergic (Booij et al., 1999) and the extrastriatal serotonergic (Koopman et al., 2012) system in vivo (Ziebell et al., 2010).

Only few studies have studied extrastriatal ^123^I-FP-CIT SERT binding in vivo in PD and DLB patients. In these studies there was evidence of lower SERT binding in the midbrain in DLB patients (Roselli et al., 2010), and higher SERT binding in the hypothalamus in PD patients compared with other forms of degenerative parkinsonism (Joling et al., 2017). Positron emission tomography (PET) and neuropathology studies, respectively, provide additional evidence for lower SERT availability in PD in the hypothalamus and thalamus (Pagano et al., 2017), and abnormal hippocampal SERT-expressing neurons in PD and DLB (Azmitia and Nixon, 2008).

In a recent comparative study using MRI-based ROIs, we found no significant differences in extrastriatal ^123^I-FP-CIT SERT binding between PD and DLB patients (Joling et al., 2018). However, because of a lack of controls with an MRI brain scan for co-registration purposes in that particular study, we could not compare SERT binding between controls and the two patient groups. To gain therapeutic and prognostic insights, it is essential to know whether there is indeed evidence of a loss of SERT-expressing neurons relative to controls in both diseases. In the present study we therefore used an already established processing method that does not require MRI scans for co-registration (Joling et al., 2017; Vriend et al., 2014; Vriend et al., 2014), to be able to compare DAT and SERT binding in carefully matched PD and DLB patients and healthy controls.

Based on the abovementioned literature, we expected to find lower striatal DAT availability in both PD and DLB patients. In addition, we expected lower extrastriatal SERT availability in the hypothalamus, thalamus and hippocampus in PD and DLB than in healthy controls.

## 2 Materials and methods

### 2.1 Participants

Patients included in this cross-sectional retrospective study were either examined at the outpatient clinic for movement disorders (PD patients), or at the Alzheimer Center (DLB patients; Amsterdam Dementia Cohort (van der Flier et al., 2014)), both part of the department of Neurology at the VU University Medical Center (VUmc; Amsterdam, the Netherlands), between November 2009 and November 2015. The patients were clinically diagnosed by a multidisciplinary team, including neurologists, according to the UK PD Society Brain Bank criteria for PD patients (Gibb and Lees, 1988; Hughes et al., 1992), or the McKeith criteria for DLB patients (McKeith et al., 2005). All patients provided informed consent to enter their clinical and imaging data, obtained as part of routine patient care, in a pseudonymised database for research purposes. This procedure was approved by the local Medical Ethics Committee of the VUmc. Sixteen healthy controls were recruited by the department of Radiology and Nuclear Medicine of the VUmc between June 2007 and July 2008, and their ^123^I-FP-CIT SPECT scans were also used in a previous study (Vriend et al., 2014). We carefully matched PD and DLB patients from the database for age and gender to the 16 healthy controls, creating three equally sized groups. Selected patients were not on serotonin reuptake inhibitors (SRIs), because this type of medication blocks the SERT (Booij et al., 2007). We performed this selection blinded for ^123^I-FP-CIT scan outcome.

### 2.2 Clinical characteristics

For the PD patients we defined disease duration as the time between the approximate self-reported onset of motor symptoms and the date of the ^123^I-FP-CIT SPECT scan. For DLB patients disease duration was defined as the time between the approximate self-reported initiation of either motor- or cognitive symptoms and the ^123^I-FP-CIT SPECT scan date. In the PD patients, severity of motor symptoms was rated using the Unified Parkinson's Disease Rating Scale, motor section (UPDRS-III) (Fahn and Elton, 1987), and disease severity using the (Hoehn and Yahr, 1967) staging system. In the DLB patients, the presence of motor symptoms was registered dichotomously. The healthy controls were not subjected to clinical scales.

### 2.3 ^123^I-FP-CIT SPECT–image acquisition and pre-processing

We acquired and pre-processed the ^123^I-FP-CIT SPECT scans as reported previously (Vriend et al., 2014). In summary, an intravenous bolus injection of approximately 185 MBq ^123^I-FP-CIT (specific activity >185 MBq/nmol; radiochemical purity >99%; produced as DaTSCAN according to GMP criteria at GE Healthcare, Eindhoven, The Netherlands) was given approximately 3 h before start of the acquisition of the scans. Subsequently static images were taken for 30 min using a single dual-head gamma camera (E.Cam; Siemens, Munich, Germany) equipped with a fan-beam collimator. The scans were acquired with a voxel size of 3.9 mm^3^, and a pixel matrix of 128 × 128. During pre-processing the scans were resliced to 2 mm3 (dimensions: 79 × 79 × 78 mm), conform Vriend et al. (Vriend et al., 2014). We used Chang's attenuation correction (Chang, 1978), with an attenuation coefficient of 0.15, for all images. Hereafter, we reoriented and normalised the reconstructed images to Montreal Neurological Institute (MNI) space in Statistical Parametric Mapping 12 software (SPM 12; Wellcome Trust Centre for Neuroimaging, London, UK) using a standardized in-house ^123^I-FP-CIT SPECT template as described previously (Vriend et al., 2014). The scans of the control subjects were obtained in the beginning of the period in which the initial scans of the patients were obtained. During the whole period in which the SPECT scans were acquired no software or hardware enhancements of the SPECT system, which may have influenced the quantification of the SPECT data, were performed.

### 2.4 ^123^I-FP-CIT SPECT–image analysis

#### 2.4.1 Region of interest (ROI) analyses

As reported previously (Joling et al., 2017), we established masks for the ROIs in MNI space from the WFU Pickatlas (Version 3.0.5; Wake Forest University, Winston-Salem, NC, USA). For the DAT-rich striatum we used the bilateral caudate head and the bilateral putamen from the Automated Anatomical Labelling (AAL) atlas. We adapted the putamen in this atlas according to an earlier published method to obtain masks of the bilateral posterior putamen (Vriend et al., 2014). We derived the bilateral SERT-rich extrastriatal ROI masks for thalamus and hippocampus also from the AAL atlas. For the hypothalamus, we defined its outlines on the Talairach Daemon (TD) Brodmann area + atlas which is implemented within the WFU Pickatlas. Because of its small dimensions, this mask was dilated to twice its original size.

We calculated specific to non-specific binding ratios in DAT- and SERT-rich regions. For this we used the non-specific binding in the cerebellum as a reference (REF; WFU Pickatlas, AAL; bilateral Crus 2), since it is relatively free from DAT and SERT (Kish et al., 2005), using the following formula in SPM 12: [Binding ratio = (ROI-REF)/REF], representing the non-displaceable binding potential (BP_ND_) (Innis et al., 2007).

#### 2.4.2 Voxel-based analyses

The ROI-based analysis yields an average binding ratio for the whole ROI. To obtain additional information, we therefore also performed voxel-based analyses of variance with age as a nuisance covariate in SPM 12 on the ROIs that showed between group differences in the ROI-based analysis. We used the formula [(voxel – REF)/REF] to adjust all voxels in the ^123^I-FP-CIT SPECT scan to the mean binding in the cerebellar reference region, making it possible to compare each voxel in the ROI between the groups. For each relevant ROI we placed an explicit mask in which we performed the voxel-based analysis. The masks were the same as in the ROI-based analysis. Statistical threshold was set to *P* < .05, Family-Wise Error corrected for multiple comparisons.

### 2.5 Statistics

We assessed normality of the data by plotting histograms, examining Q-Q plots, and using Kolmogorov-Smirnov tests for normality. For data that did not approximate a normal distribution we used non-parametric Kruskal-Wallis tests. Ageing effects on ^123^I-FP-CIT binding have been reported previously, both for striatal DAT as well as extrastriatal SERT binding (Varrone et al., 2013; Koch et al., 2014); therefore we performed analyses of covariance (ANCOVA) with age as nuisance covariate on both healthy controls versus PD patients and healthy controls versus DLB patients. We verified that assumptions for analysis of covariance were met; including homogeneity of the variances and regression slopes. We reported effect sizes as omega squared (*ω*^*2*^), where we considered 0.01, 0.06, 0.14 as small, medium and large effect sizes, respectively (Kirk, 1996).

To correct for multiple testing, we applied Simple Interactive Statistical Analysis (SISA; http://www.quantitativeskills.com/sisa/calculations/bonhlp.htm) to calculate corrected *P*-values (*P*_corr_). This tool uses the mean association between variables that are mutually correlated in four striatal ROIs (*r* = 0.89 for PD, *r* = 0.91 for DLB) and five extrastriatal ROIs (*r* = 0.91 for PD, *r* = 0.73 for DLB) for the alpha correction. This resulted in statistical thresholds of *P*_corr_ = 0.043 (PD) and *P*_corr_ = 0.034 (DLB) for striatal ROIs, and *P*_corr_ = 0.033 (PD) and *P*_corr_ = 0.032 (DLB) for extrastriatal ROIs. All statistical analyses were conducted in SPSS 22 (IBM Corp, Armonk, NY).

## 3 Results

### 3.1 Characteristics

The clinical characteristics of the participants are summarised in Table 1. PD patients did not differ significantly in age from the healthy controls (*T*(31) = −0.036, *P* = .971), whereas DLB patients were slightly, but significantly, older than the healthy controls (*T*(31) = −2.456, *P* = .023). Disease duration was not significantly different between the PD (median 2.5 years) and DLB (median 3.0 years) patients (U = 119.000, *P* = .984). As expected, MMSE scores were higher in PD patients than in DLB patients (*U* = 17.500, *P* < .001).

**Table 1:** Clinical characteristics.

	HC	PD	DLB	Statistics
*N*	16	16	16	
*Gender (f/m)*	8/8	8/8	8/8	
*Age at DAT (yr), mean (SD)*	57.5 (10.1)	57.6 (10.2)	64.4 (4.9)	
*PD*_*vs*_*HC*				*T*(31) = −0.036, *P* = .971
*DLB*_*vs*_*HC*				*T*(31) = −2.456, *P* = .023
*Disease duration, median (IQR)*	N/A	2.5 (3.8)	3.0 (2.0)	*U* = 119.000, *P* = .984
*MMSE, median (IQR)*	N/A	28.5 (1.0)	22.5 (7.0)	*U* = 17.500, *P* ≤.001
*UPDRS-III, mean (SD)*	N/A	25.6 (12.3)	N/A	
*H&Y, median (IQR)*	N/A	2.00 (0.0)	N/A	

Values given are mean ± standard deviation, unless otherwise specified; PD_vs_HC *t*-test on age between PD and HC. DLB_vs_HC *t*-test on age between DLB and HC. MMSE, Mini Mental State Examination; UPDRS III, Unified Parkinson's Disease Rating Scale: motor evaluation; H&Y, Hoehn and Yahr disease stage; HC, healthy controls; PD, Parkinson's disease; DLB, dementia with Lewy bodies; N/A, not available; *df*, degrees of freedom; *F*, Analysis of variance *F*-statistic; *U*, Mann-Whitney *U*-statistic.

### 3.2 Region of interest (ROI) based ^123^I-FP-CIT analyses

#### 3.2.1 Striatal ROIs

**Fig. 1:**
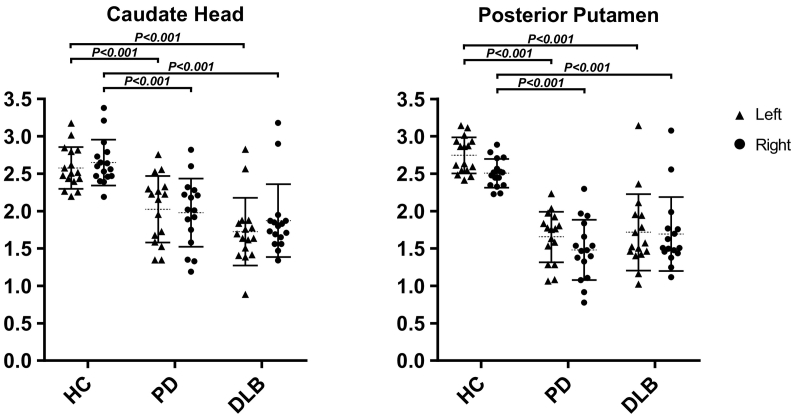
Mean specific to non-specific binding ratios in striatal ROIs. HC, healthy controls; PD, Parkinson's disease; DLB, dementia with Lewy bodies; error bars represent the standard deviation (SD).

PD patients had lower ^123^I-FP-CIT binding ratios than healthy controls for the bilateral caudate head (left: *F*(1,29) = 28.778, *P* < .001, *ω*^*2*^ = 0.35; right: *F*(1,29) = 35.338, *P* < .001, *ω*^*2*^ = 0.42) and the bilateral posterior putamen (left: *F*(1,29) = 107.531, *P* < .001, *ω*^*2*^ = 0.77; right: *F*(1,29) = 87.525, *P* < .001, *ω*^*2*^ = 0.72). Similarly, DLB patients had lower ^123^I-FP-CIT binding ratios than healthy controls in the bilateral caudate head (left: *F*(1,29) = 28.241, *P* < .001, *ω*^*2*^ = 0.31; right: *F*(1,29) = 18.811, *P* < .001, *ω*^*2*^ = 0.26) and the bilateral posterior putamen (left: *F*(1,29) = 39.910, *P* < .001, *ω*^*2*^ = 0.48; right: *F*(1,29) = 26.882, *P* < .001, *ω*^*2*^ = 0.38). See Fig. 1.

#### 3.2.2 Extrastriatal ROIs

**Fig. 2:**
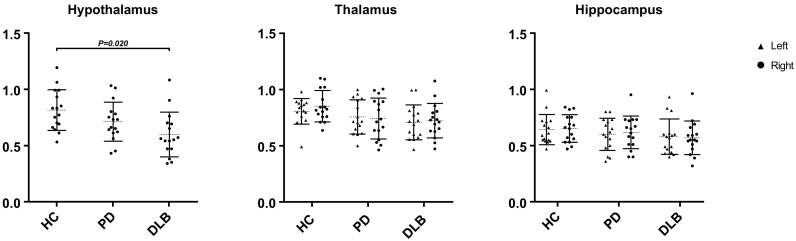
Mean specific to non-specific binding ratios in extrastriatal ROIs. HC, healthy controls; PD, Parkinson's disease; DLB, dementia with Lewy bodies; error bars represent the standard deviation (SD).

DLB patients had lower ^123^I-FP-CIT binding ratios for the hypothalamus (*F*(1,29) = 6.059, *P* = .020, *ω*^*2*^ = 0.12) than healthy controls. We did not find significant differences in ^123^I-FP-CIT binding ratios in the bilateral thalamus or hippocampus of both PD and DLB compared with the healthy controls. See Fig. 2. A recent study in 103 healthy controls showed higher ^123^I-FP-CIT binding in the thalamus in women than in men (Koch et al., 2014). In the present study, post-hoc analyses did not show statistically significant sex differences when comparing the SERT ROIs between males and females both within the patient and the control group (data not shown). This discrepancy may be caused by the large difference in the number of subjects studied.

### 3.3 Voxel-based ^123^I-FP-CIT analyses

#### 3.3.1 Striatal ROIs

**Fig. 3:**
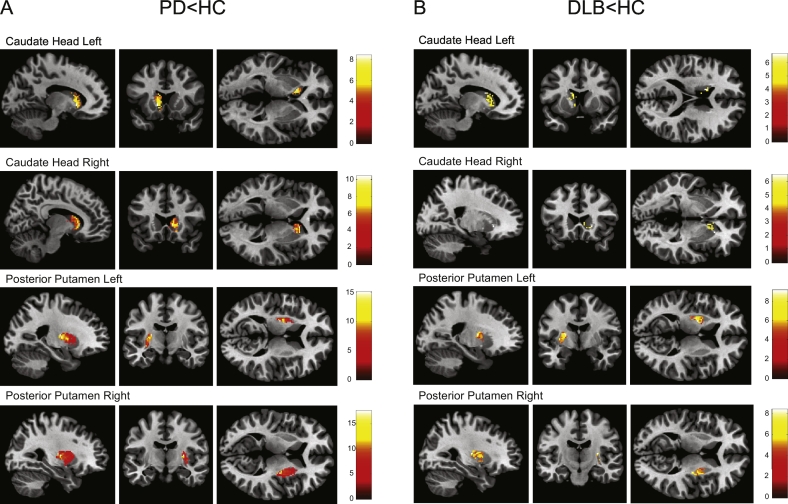
Voxel-based analyses of covariance of ^123^I-FP-CIT binding in striatal areas. *A)* PD lower than HC; *B)* DLB lower than HC. No significant clusters with increased DAT or SERT binding were found in PD or DLB compared with HC. HC, healthy controls; PD, Parkinson's disease; DLB, dementia with Lewy bodies.

The voxel-based analyses for the caudate head and posterior putamen confirmed the findings in the ROI-based analyses. See Table 2 and Fig. 3.

**Table 2:** Voxel-based analysis.

Region	Contrast	K_e_	*P*_*FWE*_ Cluster	*T*	x/y/z (mm)
Caudate head left	PD < HC	175	<0.001	8.41	−14/16/0
	DLB < HC	181	<0.001	6.70	−14/10/18
		4	0.023	6.04	−8/16/−8
Caudate head right	PD < HC	301	<0.001	10.38	10/20/2
	DLB < HC	92	<0.001	6.50	20/22/−2
		5	0.007	5.18	10/10/14
		5	0.007	5.17	4/10/−4
Posterior putamen left	PD < HC	568	<0.001	15.16	−24/−10/12
	DLB < HC	324	<0.001	9.17	−22/−4/10
Posterior putamen right	PD < HC	618	<0.001	17.62	28/−10/8
	DLB < HC	221	<0.001	8.40	28/−14/10

Analyses of covariance between PD and healthy controls (PD < HC) and DLB and healthy controls (DLB < HC), degrees of freedom: 1,29; Ke, cluster extend in number of voxels; *P*_FWE_, family-wise error corrected P-values; *F, F*-statistic; x/y/z, location of significantly most different between groups cluster from midpoint in millimetre in Montreal neurological Institute space; HC, Healthy Controls; PD, Parkinson's disease; DLB, dementia with Lewy bodies.

#### 3.3.2 Extrastriatal ROIs

In the extrastriatal ROIs we did not find a significant difference ^123^I-FP-CIT binding between PD or DLB patients and healthy controls in thalamus, hypothalamus or hippocampus.

## 4 Discussion

In this retrospective cross-sectional study we examined ^123^I-FP-CIT binding in both PD and DLB in comparison to healthy controls, as a proxy for the integrity of the striatal dopaminergic system and the extrastriatal serotonergic system in vivo. With a median disease duration of 2.5 years for the PD patients and 3.0 years for the DLB patients, the PD group can be considered representative of the early disease stages. In accordance with the results of previous studies (Walker et al., 2004; O'Brien et al., 2004), we observed significantly lower ^123^I-FP-CIT binding ratios in both the bilateral caudate head and the posterior putamen of PD and DLB patients as compared with healthy controls. The effect sizes—expressed as *ω*^*2*^—of these striatal differences were large for both PD and DLB in all striatal regions. In the extrastriatal areas, we observed lower ^123^I-FP-CIT binding ratios in the hypothalamus of DLB patients with an *ω*^*2*^ of 0.12, which can be considered a medium effect size. In the voxel-based analysis, we corroborated the striatal loss of ^123^I-FP-CIT binding, but we did not find significant differences in extrastriatal ^123^I-FP-CIT binding.

Potentially lower SERT availability in DLB, as demonstrated in the present study, is in line with previous findings. For example, significant morphological differences of SERT-positive prefrontal cortical neurons have been reported between healthy controls and PD and DLB patients (Azmitia and Nixon, 2008). A histopathological study in DLB has shown a loss of serotonergic neurons in the dorsal and median raphe nuclei (Benarroch et al., 2007), and the median raphe nuclei have been shown to project to the hypothalamus (Hornung, 2003). A single ^123^I-FP-CIT SPECT study demonstrated lower midbrain SERT binding in both PD and DLB than in healthy controls, the loss of SERT being more pronounced in DLB than in PD (Roselli et al., 2010). Additionally, a recent meta-analysis of studies in PD patients revealed a loss of thalamic and hypothalamic SERT measured with ^11^C-3-amino-4-(2-dimethylaminomethylphenylsulfanyl)-benzonitrile (^11^C-DASB), a SERT-selective PET tracer (Pagano et al., 2017).

The studies included in this meta-analysis were mainly conducted in advanced PD patients (average disease duration 7.4 years), whereas our PD patients had a much shorter disease duration (average 2.5 years). This difference in disease duration may explain why differences in SERT binding were less profound in the present study in early-stage PD patients, and why we failed to find lower binding in the thalamus in PD and DLB, and in the hypothalamus in PD. Noticeably, the definition of disease duration was reported for only 7 of the 20 reviewed studies in the meta-analysis. They used the same method as we did by taking the initiation of motor symptoms as initial moment of the disease. However, since disease duration might also be defined as years after diagnosis, actual differences in disease duration may even have been larger.

In a previous comparative ^123^I-FP-CIT SPECT study, we found that PD patients had higher hypothalamic SERT availability than patients with progressive supranuclear palsy (PSP) and the parkinsonian form of multiple-system atrophy (MSA-P) (Joling et al., 2017). Similar to DLB, PSP and MSA-P are both diseases with a more rapid rate of neurodegeneration than PD. Shannak and colleagues reported interindividual differences in PD patients in levels of hypothalamic serotonin compared with healthy controls, with several patients showing normal serotonin levels (Shannak et al., 1994). Our current observation of reduced hypothalamic SERT availability in the more rapidly progressing DLB patients, but not in PD patients, would seem to be in line with Shannak et al., and with our previous observations in PSP and MSA-P.

Serotonergic terminals in the hypothalamus play an important, often stimulating, role in autonomic functions that are involved in stress responses (Jorgensen, 2007), and the prevalence of orthostatic hypotension, for example, is reportedly higher in DLB than in PD patients and healthy controls (Thaisetthawatkul et al., 2004; Andersson et al., 2008). Quite possibly, therefore, neurodegeneration of SERT-expressing terminals in the hypothalamus could contribute to non-motor symptoms such as autonomic dysfunction. Another finding that further supports the involvement of the hypothalamus in DLB is a comparative MRI study in which DLB patients had more hypothalamic atrophy than patients with Alzheimer's disease (Whitwell et al., 2007). However, the scans of the DLB patients in that study were not compared with scans of healthy controls. Since we were likewise unable to compare ROI volumes between patients and healthy controls using MRI, this speculation needs further attention in future studies.

In another previous comparative ^123^I-FP-CIT SPECT study we found no differences in extrastriatal SERT availability between PD and DLB (Joling et al., 2018). However, to establish the presence of neurodegeneration of SERT-expressing terminals in both diseases, a comparison of both patient groups with healthy controls was essential, which is why we performed the present study. As a consequence of the lack of an MRI scan in the healthy controls, the choice of ROIs in the present study was different from our previous study in which we used an amygdala mask and had no hypothalamus mask (Joling et al., 2018). In contrast to an MRI-based approach, which allows a delineation of subcortical areas in individuals (e.g. using FreeSurfer), this is not possible when using only an ^123^I-FP-CIT SPECT scan with a limited spatial resolution. Since ^123^I-FP-CIT binding in the amygdala has never been validated properly, and binding ratios in this region are relatively low, the lack of an MRI scan for exact co-registration would most likely lower accuracy. We therefore omitted this mask as a ROI in this study. Given the previous evidence of lower SERT availability in the hypothalamus (Pagano et al., 2017), we used a standard mask of the hypothalamus as a ROI conform our previous study (Joling et al., 2017).

A limitation of this study is the lack of MRI scans in the healthy controls, making a personalised MRI-based ROI approach impossible. It also impedes on the possibility to assess brain atrophy in patients compared with healthy controls. We therefore cannot exclude that loss of ^123^I-FP-CIT binding is (partly) due to atrophy. However, the current atlas-based method has already been successfully applied in previous studies (Joling et al., 2017; Vriend et al., 2014). Moreover, although an effect on binding ratios due to differences of cerebral blood flow cannot entirely be excluded, however, we considered this effect as being unlikely since the scans were obtained at 3 h after injection of the radiotracer. For the healthy controls UPDRS motor scores were not available. This is also a limitation, since it could contain information about possible initial subtle motor symptoms in these subjects. Another limitation is its relatively small sample size, but we believe this limitation was mitigated by including equal-sized groups of PD and DLB patients and healthy controls that were matched for disease duration and sex. Although we tried to match the patients to the healthy controls for age, DLB patients were still somewhat older than the healthy controls and PD patients, possibly introducing an age effect in the loss of ^123^I-FP-CIT binding. Consequently, we used age as a covariate in the analyses to correct for potential age effects on binding ratios in individual ROIs. In the present study we did not use a selective SERT tracer to assess SERT binding in-vivo. This could be another limitation, although previous studies in healthy controls showed that ^123^I-FP-CIT binding in the diencephalon and midbrain could be blocked or displaced by an SSRI (Ziebell et al., 2010; Booij et al., 2007), and the affinity of the radiotracer for the DAT is higher than that for the SERT (Abi-Dargham et al., 1996), we cannot exclude that extrastriatal ^123^I-FP-CIT binding represents partly binding to the DAT. Lastly, the extrastriatal result in the hypothalamus differed between the ROI and the voxel-based analyses. This is probably due to a technical difference, where in the voxel-based analysis no single voxel survived the Family Wise Corrected significance threshold value, which is in line with the medium effect size of this finding.

In conclusion, this study shows that, in addition to the degeneration of dopaminergic terminals in early-stage PD and in DLB patients, there is loss of serotonergic terminals in the hypothalamus in DLB patients compared with healthy controls, but not in other SERT-rich ROIs in PD and DLB. This observation adds to the growing body of evidence that PD and DLB are not merely dopaminergic diseases, thereby providing additional clinicopathological insight that may inspire further research into pathophysiology of extrastriatal areas.

## 5 Disclosure statement

MJ: salary was paid by a research grant from GE healthcare (paid to the institution). JB: received research grants from GE Healthcare (paid to the institution). OAVDH: is co-applicant of research grants obtained from GE Healthcare (paid to the institution). HWB: is co-applicant of research grants obtained from GE Healthcare (paid to the institution) CV, PGHMR, JJZ, AWL: declare no conflicts of interest.
